# Oxidative Stress in Alzheimer's Disease: *In Vitro* Therapeutic Effect of Amniotic Fluid Stem Cells Extracellular Vesicles

**DOI:** 10.1155/2020/2785343

**Published:** 2020-10-24

**Authors:** Martina Gatti, Manuela Zavatti, Francesca Beretti, Daniela Giuliani, Eleonora Vandini, Alessandra Ottani, Emma Bertucci, Tullia Maraldi

**Affiliations:** ^1^Department of Biomedical, Metabolic and Neural Sciences, University of Modena and Reggio Emilia, Modena 41125, Italy; ^2^Department of Medical and Surgical Sciences for Mothers, Children and Adults, University of Modena and Reggio Emilia, Azienda Ospedaliero Universitaria Policlinico, Via Del Pozzo 71, 41124 Modena, Italy

## Abstract

Alzheimer's disease (AD) is characterized by abnormal protein aggregation, deposition of extracellular *β*-amyloid proteins (A*β*), besides an increase of oxidative stress. Amniotic fluid stem cells (AFSCs) should have a therapeutic potential for neurodegenerative disorders, mainly through a paracrine effect mediated by extracellular vesicles (EV). Here, we examined the effect of EV derived from human AFSCs (AFSC-EV) on the disease phenotypes in an AD neuron primary culture. We observed a positive effect of AFSC-EV on neuron morphology, viability, and A*β* and phospho-Tau levels. This could be due to the apoptotic and autophagic pathway modulation derived from the decrease in oxidative stress. Indeed, reactive oxygen species (ROS) were reduced, while GSH levels were enhanced. This modulation could be ascribed to the presence of ROS regulating enzymes, such as SOD1 present into the AFSC-EV themselves. This study describes the ROS-modulating effects of extracellular vesicles alone, apart from their deriving stem cell, in an AD *in vitro* model, proposing AFSC-EV as a therapeutic tool to stop the progression of AD.

## 1. Introduction

Neurodegenerative diseases are characterized by progressive damage in neural cells which leads to compromised motor or cognitive functions. Alzheimer's disease (AD) is the sixth leading cause of death in the United States, and it is the most widespread neurodegenerative disorder, which significantly affects people's daily lives [1]. The pathophysiology of AD is mainly associated with the extracellular deposition of amyloid beta (A*β*) plaques and the accumulation of intracellular tau neurofibrillary tangles (NFT) [2]. The mechanisms behind A*β* neurotoxic action could be several; many of which flow into the reactive oxygen species (ROS) generation. For example, A*β* plaques can reduce calcium ion storage in endoplasmic reticulum, then resulting in cytosolic Ca^2+^ overload. As a consequence of cytosolic Ca^2+^ increase, endogenous glutathione (GSH) levels are reduced and ROS are overaccumulated inside the cells [3]. In addition, A*β* proteins can directly cause free radical formation *via* the activation of NADPH oxidase [4]. Indeed, ROS are chemically reactive molecules that are involved in the pathogenesis of neurodegenerative diseases. Although it is not yet understood whether ROS could be the triggering factor in neurodegenerative diseases, they probably are to be considered exacerbating players in the disease progression through oxidative damage [5, 6]. Of note is that neuronal cells are particularly vulnerable to oxidative damage because of their high polyunsaturated fatty acid content in membranes, high oxygen consumption, and weak antioxidant defense [7].

Moreover, ROS can regulate JNK/stress-activated protein kinase pathways. The activation of these cascades is linked to the hyperphosphorylation of tau proteins and A*β*-induced cell death [8]. The pathogenesis of several neurodegenerative disorders, such as AD and Parkinson's disease, is associated with the accumulation of misfolded proteins. Inflammatory response in the brain can be activated to counteract the aggregation of these modified proteins, while inducing a marked ROS release and subsequent oxidative stress (OS) [9, 10]. Therefore, a vicious circle, in which the ROS level increases, seems to be triggered. Considering the pivotal roles of OS in neurodegenerative diseases, the regulation of ROS levels could be a promising target to slow down neurodegeneration and alleviate associated symptoms. However, most of the antioxidants which have been studied, proved scarcely successful in clinical trials, which may be due to their scarce distribution and inherent difficulties to cross the blood brain barrier and reach the target sites. Despite the evidence that different classes of antioxidants are neuroprotectants *in vitro*, the clinical data is not consistent [11]. Based on these considerations, finding a way to combine neuroprotective, anti-inflammatory, and endogenous antioxidant manipulation properties may hold great promise to counteract AD symptoms.

Mesenchymal stem cells (MSCs) and, in particular, MSC-derived extracellular vesicles (EV), could be proposed as a strategic approach to contrast this pathology. Indeed, MSC-EV—carrying lipids/proteins/enzymes/microRNAs endowed with anti-inflammatory, A*β* degrading systems, and neurotrophic activities [12]—could be the therapeutic tool combining different properties able to restore the synaptic function, prevent neuronal death, and slow down memory impairment in AD.

Several demonstrations of the effect of MSC-EV on lowering AD markers are present in the literature on the *in vitro* AD cell models. The exposure to MSC-EV was first investigated in a cellular model of AD by using EVs isolated from adipose tissue MSCs. The incubation of MSC-EVs to N2A neuroblastoma cells reduced both secreted and intracellular A*β* peptide levels [13]. Similarly, more recently MSC transwell coculture with rat neurons exposed to soluble oligomers of the A*β* peptide (A*β*Os) induced internalization and degradation of A*β*Os, release of extracellular vesicles containing active catalase, and selective secretion of interleukin-6, interleukin-10, and vascular endothelial growth factor to the medium [14].

Amniotic fluid stem cells (AFSCs) are a feasible variety of MSC for experimentation purposes, owing to their pluri/multipotency, abundance, and minimal ethical considerations. We recently reported that AFSC secretory factors have antiapoptotic and anti-inflammatory effects [15, 16]. Although the paracrine function of stem cells is reported to have a therapeutic potential in many neurological disorders, the effects of AFSC-derived extracellular vesicles (AFSC-EV) on the cellular phenotypes of AD have not yet been investigated. A primary culture of neurons, derived from 5xFAD mice [17, 18], an animal model of AD, was here employed to investigate the effect of an enrichment in extracellular vesicles obtained from human AFSCs on morphological features, apoptosis/viability parameters, and AD markers. We focused our attention on the EV-modulation of ROS levels as pivotal key to counteract neurodegenerative diseases.

## 2. Materials and Methods

### 2.1. Amniotic Fluid Collection

The AFSCs were obtained from amniotic fluids collected from 4 healthy pregnant women at the 16th week of gestation who underwent amniocentesis for maternal request (not for foetal anomalies) at the Unit of Obstetrics & Gynecology, IRCCS-ASMN of Reggio Emilia and at the Policlinico Hospital of Modena (Italy). The amniocentesis was performed under continuous ultrasound guidance, in a sterile field, with 23-Gauge needles. The risks related to the procedure and the purpose of the study were explained to all patients before the invasive procedure and the ob-gyn specialist collected a signed consent before starting the exam (protocol 2015/0004362 of 02.24.2015 and protocol 360/2017 dated 12.15.2017 approved by Area Vasta Emilia Nord). For this study, supernumerary (unused) flask of AF cells, cultured in the Laboratory of Genetics of TEST Lab (Modena, Italy) for 2 weeks, was used.

### 2.2. Adult Human Tissue Isolation and Cell Culture

AFSCs were isolated as previously described [19]. Human amniocentesis cultures were harvested by trypsinization and subjected to c-kit immunoselection by MACS technology (Miltenyi Biotec, Germany). AFSCs were subcultured routinely at 1 : 3 dilution and not allowed to grow beyond the 70% of confluence. AFSCs were grown in culture medium (*α*MEM) supplemented with 20% fetal bovine serum (FBS), 2 mM L-glutamine, 100 U/ml penicillin, and 100 *μ*g/ml streptomycin (all from EuroClone Spa, Milano, Italy).

### 2.3. Extracellular Vesicle Isolation from Conditioned Medium

AFSCs were grown in 75 cm^2^ flask until subconfluence (around 1 × 10^6^ cells). Before extracellular vesicle extraction, the cells were maintained for 4 days in 10 mL culture medium deprived of FBS in order to exclude the contamination by extracellular vesicles comprised into FBS solution. The secreted part of conditioned medium (CM) was then concentrated up to 2 mL by using centrifugal filter units with 3 K cutoff [15]. Then, the concentrated CM was treated with total exosome isolation solution from cell culture media (Invitrogen, Life Technologies, CA. USA), according to the manufacturer's instructions. The pellet, enriched in exosomes although it can be not a pure extraction, was collected and quantified by the Bradford method. To obtain a sample for Western blot analysis, the pellet was resuspended in lysis buffer. We previously showed morphological characterization of extracellular vesicles by electron microscopy [15].

The surnatants deriving from the isolation protocol (-EV) were collected as well. The neuroblastoma cell line SH-SY 5Y, cultured in 1 : 1 DMEM (EuroClone Spa, Milano, Italy)/F12 (EuroClone Spa, Milano, Italy), 1% fetal bovine serum (FBS) (Microgem, Naples, Italy), 2 mM glutamine, 100 U/ml penicillin and 100 *μ*g/ml streptomycin, 1 mM Not-Essential Amino Acids (all from EuroClone Spa, Milano, Italy), was exposed to 10 *μ*M A*β* 1-42 (Anaspec Inc., California, USA) for 24 h in order to test EV and -EV in a simple AD *in vitro* model. The same protein concentration of +EV and –EV was added to neuronal cells 3 days before A*β* treatment.

### 2.4. Animals

5xFAD mice cooverexpress a triple-mutant human amyloid precursor protein (APP) (Swedish mutation: K670N, M671L; Florida mutation: I716V; London mutation: V717I) and a double-mutant human presenilin 1 (PS1) (M146L and L286V mutations) transgenes under the transcriptional control of the neuron specific Thy-1 promotor. Progenitors were purchased from Jackson Laboratories, Bar Harbor, and 5xFAD line was maintained by crossing hemizygous 5xFAD mice with B6SJL/J breeder. Mice were kept in conditioned rooms with stable temperature (22 ± 1°C) and humidity (60%), on a light/dark cycle of 12 hours, with food and water *ad libitum*. All animal procedures were approved by the Committee on Animal Health and Care of the University of Modena and Reggio Emilia (protocol number: 974/2016-PR del 13-10-2016) and conducted in accordance with National Institutes of Health guidelines.

For genotyping procedure, APP and PS1 lines were analyzed as previously reported (Gordon et al. 2001). Tail samples from WT and 5xFAD mice were examined by using primers supplied by Jackson Laboratories to identify either the mutation or the wild-type allele with the polymerase chain reaction (PCR): forward, 5′-AGGACTGACCACTCGACCAG-3′ and reverse, 5′-CGGGGGTCT AGTTCTGCAT-3′ for APP transgenes; forward, 5′-CTAGGCCACAGAATTGAAAGATCT-3′ and reverse 5′-GTAGGTGGAAATTCTAGCATCA-TCC-3′ for positive control; forward, 5′-AAT AGA GAA CGG CAG GAG CA-3′ and reverse 5′-GCC ATG AGG GCA CTA ATC AT-3′ for hPRES transgene. DNA was polymerized at 94°C for 3 min; 35 cycles of 94°C for 15 s; 54°C for 1 min; 72°C for 1 min; and 72°C for 2 min; stored at 4°C. The PCR products were run on a 1% agarose gel, using ethidium bromide ultraviolet (UV) detection for the bands at 377 bp (APP transgene) or 324 bp (positive control) and 608 bp (hPRES).

### 2.5. Preparation of Primary Cultured Cortical Neurons

Cortical and hippocampal neurons from wild type (WT) (*N* = 18) and 5xFAD (FAD) (*N* = 25) mice were collected within 24 h from birth [20]. Brains were removed, meninges were gently peel off, and cortical and hippocampal tissue dissected. Tissues were incubated in a buffer solution containing HBSS (Hanks' Balanced Salt Solution, GIBCO, Milan, Italy) with BSA 0.3% (Microgem Srl, Naples, Italy) and trypsin 0.025% (EuroClone, Milan, Italy) at 37°C for 15 min. Then, tissues were triturated with a plastic 1000 *μ*l pipette tip in a HBSS buffer containing 0.004% deoxyribonuclease I (DNAseI, Sigma Aldrich) and 10% fetal bovine serum (FBS, Microgem Srl). After centrifugation at 500 g for 5 min, cells were suspended in Neurobasal medium supplemented with 2% B27 (GIBCO, Thermo Fisher Scientific, Monza, Italy), 2 mM glutamine, 100 U/ml penicillin and, 100 *μ*g/ml streptomycin (EuroClone) and plated on poly-L-lysine (Merck Millipore, Milan, Italy) coated plastic or coverslips at 0.5 x10^6^ cells/cm^2^. Cells were maintained at 37°C and 5% CO_2_. After 48 h, cells were treated with 5 *μ*M cytosine *β*-D-arabinofuranoside (Sigma Aldrich) for 24 h.

For *in vitro* experiments, 10 *μ*g of extracellular vesicles, obtained from the two human samples of AFSCs, resuspended in PBS, were added to 1 × 10^6^ cells at DIV (days *in vitro*) 3 for 7 up to 14 days.

Wortmannin (Sigma Aldrich) treatment was performed for 2 h prior the EV exposure.

### 2.6. Cellular Morphology

Cellular images were acquired using EVOS XL Core Cell Imaging System (Thermo Fisher Scientific, Vantaa, Finland). Parameter and area were measured with ImageJ using image pixels as scale. Cellular elongation was calculated using the following formula:

(1) $$\begin{array}{c} \text{Cellular elongation}=\text{p}2/4\pi *\text{A} \end{array}$$

where *p* is the cellular perimeter, *π* is equivalent to 3.14, and *A* represents the cellular area.

### 2.7. MTT Assay

Primary neurons were seeded in 96-well plates in 200 *μ*l of a culture medium, 4 replicates for each condition. At the end of each experiment, 0.5 mg/ml MTT was added and incubated for 3 h at 37°C. After incubation, the medium was removed and acidified isopropanol was added to solubilize the formazan salts [21]. The absorbance was measured at 570 nm using a microplate spectrophotometer (Appliskan, Thermo-Fisher Scientific, Vantaa, Finland).

### 2.8. ROS and Glutathione Detection

To evaluate intracellular ROS levels, dichlorodihydrofluorescein diacetate (DCFH-DA) assay was performed similarly to as previously described [22]. Cells were seeded in a black 96-well plate, 4 replicates for each condition. Cell culture medium was removed, and the 5 *μ*M DCFH-DA was incubated in PBS for 30 min, at 37°C and 5% CO_2_. The cell culture plate was washed with PBS, and fluorescence of the cells was read at 485 nm (excitation) and 535 nm (emission) using the multiwall reader Appliskan (Thermo-Fisher Scientific, Vantaa, Finland). Cellular autofluorescence was subtracted as a background using the values of the wells not incubated with the probe.

Similarly, to evaluate reduced GSH levels, monochlorobimane (MCB) assay was performed as previously reported [23]. Cell culture medium was removed, and 50 *μ*M MCB was incubated in PBS for 30 min, at 37°C and 5% CO_2_. The cells were washed in PBS, and fluorescence of the cells was measured at 355 nm (excitation) and 460 nm (emission).

### 2.9. Quantification of A*β* by ELISA

Secreted amyloid beta peptide1-42 (A*β*42) from primary cultured neurons was analyzed in medium exposed to neurons by BETA-APP42 ELISA Kit (Human) (Aviva Systems Biology, San Diego, CA, USA).

Medium from 5xFAD cells treated with AFSC-EV for 7 days and control were collected and then centrifuged at 20°C 1200 rpm for 10 minutes in order to remove cellular debris.

Supernatants were concentrated about 10 times by lyophilization with LIO5P (5Pascal, MI, Italy) (Sun et al. 2014). Freeze-dried powder obtained from 6 ml of medium exposed to neurons was rehydrated with 600 *μ*l of purified water and stored at +4°C until test. Concentrated media were used for the ELISA assay.

A*β* ELISA was performed according to the manufacturer's protocol of ELISA kit (Aviva Systems Biology, San Diego, CA, USA).

### 2.10. Cellular Extracts Preparation

Cell extracts were obtained as previously described [24]. Briefly, subconfluent cells were extracted by addition of lysis buffer (20 mM Tris-Cl, pH 7.0; 1% Nonidet P-40; 150 mM NaCl; 10% glycerol; 10 mM EDTA; 20 mM NaF; 5 mM sodium pyrophosphate; and 1 mM Na_3_VO_4_) and freshly added Sigma Aldrich Protease Inhibitor Cocktail and para-Nitrophenylphosphate (pNPP) at 4°C for 20 min. Lysates were sonicated, cleared by centrifugation, and immediately boiled in SDS reducing sample buffer.

### 2.11. SDS PAGE and Western Blot

Whole cell lysates from primary neurons and AFSC-EV were processed as previously described [25]. Primary antibodies were raised against the following molecules: Actin, Nox4 (Sigma-Aldrich, St Louis, MO, USA), Akt, SOD1, SIRT1, gp91phox, TrxR1, TrxR2, Gpx1, LC3*β*, PARP, TIA-1 (Santa Cruz Biotechnology, CA, USA), pAkt^ser473^ (Cell Signaling Technology, MA, USA), *β*-Amyloid clone 6E10 (Bio Legend, CA, USA), pTau^ser422^ (OriGene Technologies, MD, USA), Rab5 (Lonza, SC, USA), CD9 (Life Technologies, CA, USA), CD81 (Thermo Fisher Scientific, MA, USA), Bcl-2 (Bio Source, CA, USA).

Secondary antibodies, used at 1 : 3000 dilution, were all from Thermo Fisher Scientific (Waltham, MA, USA).

### 2.12. Immunofluorescence and Confocal Microscopy

For immunofluorescence analysis, primary neurons seeded on coated coverslips were processed and confocal imaging was performed using a Nikon A1 confocal laser scanning microscope, as previously described [26].

Primary antibodies to detect *β*Tubulin III (Millipore, CA, USA), pTau^ser422^ (OriGene Technologies, MD, USA), and 6E10 (Bio Legend, CA, USA) were used following datasheet recommended dilutions. Alexa secondary antibodies (Thermo Fisher Scientific, Waltham, MA, USA) were used at 1 : 200 dilution.

The confocal serial sections were processed with ImageJ software to obtain three-dimensional projections. The image rendering was performed by Adobe Photoshop software.

For apoptosis detection, after 3 washes with 50 *μ*l of binding buffer (10 mM HEPES, pH 7.5, containing 140 mM NaCl, and 2.5 mM CaCl_2_), the specimen was incubated for 15 minutes with 50 *μ*l of double staining solution (binding buffer containing 0.25 *μ*l of annexin V-FITC and 0.25 *μ*l of propidium iodide (PI); BD Pharmingen™, Erembodegem, Belgium). Finally, the specimen was washed 5 times with 50 *μ*l of binding buffer, mounted with 15 *μ*l of binding buffer, and visualized under fluorescence microscopy [24].

### 2.13. Statistical Analysis

*In vitro* experiments were performed in triplicate. For quantitative comparisons, values were reported as mean ± SEM based on triplicate analysis for each sample. To test the significance of observed differences among the study groups, one-way ANOVA with Bonferroni post hoc test or Student *t* test (where only two samples were compared) were applied. A *p* value < 0.05 was considered to be statistically significant. FAD and FAD + EV samples were compared to WT sample and the ANOVA analysis was shown with asterisk over the column; the same analysis revealed that the comparison between FAD and FAD + EV and the significativity was shown with additional asterisks over lines. Statistical analysis and plot layout were obtained by using GraphPad Prism® release 6.0 software.

## 3. Results

### 3.1. Morphological Characterization of Cultured Neurons Exposed to AFSC-EV

**Figure 1:**
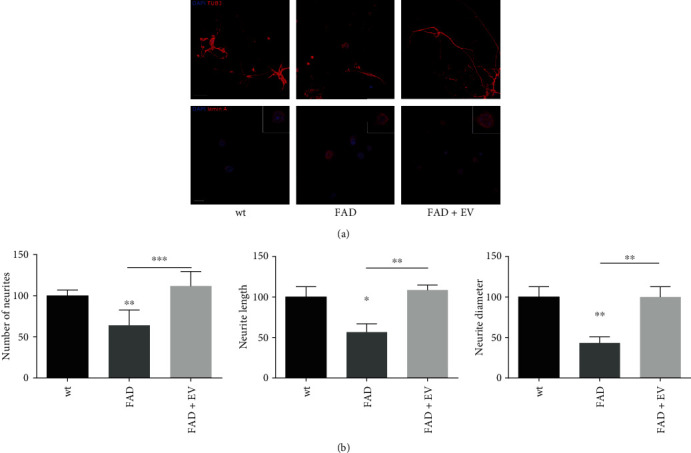
Effect of AFSC-EV supplementation on cell morphology of AD primary neurons *in vitro*. (a) Cells were incubated for 14 days with neurobasal medium 2% B27 containing AFSC-EV replaced once a week. Representative images with DAPI (blue) and *β*TubulinIII (red) or lamin A (red) signals of WT and FAD cells incubated or not with EV are shown. Scale bar = 10 *μ*m. In white squares are shown representative doubled magnification images. (b) Mean measures in percentage ± SEM of number, length, and diameter of neurites visualized in 10 fields for each condition are shown in graphs. ^∗^*p* value < 0.05; ^∗∗^*p* value < 0.01; ^∗∗∗^*p* value < 0.001.

FAD neurons appeared unhealthy, if compared to WT neurons, since neurodegeneration in FAD neurons was prominent, as indicated by shrunken nuclear envelope (Figure 1(a) lower images) and neurite deterioration (Figure 1(a) upper images), as shown in graphs displaying number, length, and thickness (Figure 1(b)). The effect of 14 days EV treatment was to counteract the nuclear envelope modification and to avoid neurite loss, since the measures of neurite parameters were brought back similar to the WT ones. Similar results were obtained in all the experiments shown here below, despite individual variability due to AFSC samples obtained from different women.

### 3.2. ROS Modulation by AFSC-EV in FAD Neurons

**Figure 2:**
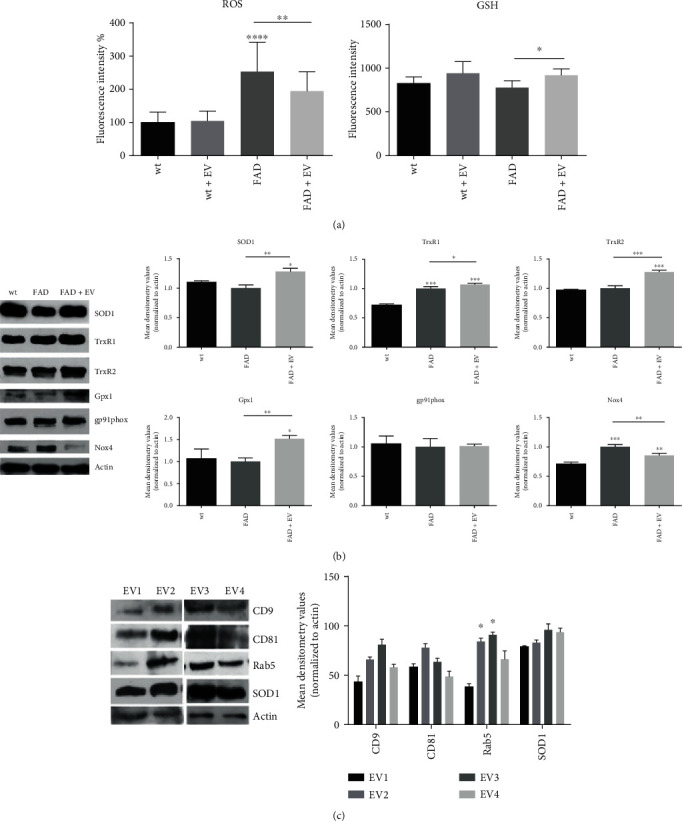
Effect of AFCS-EV supplementation on ROS modulation in AD primary neurons. (a) ROS and GSH content was measured with fluorescent probes, as described in Section 2.8, after 7 days of AFSC-EV exposure. ^∗^*p* value < 0.05; ^∗∗^*p* value < 0.01; ∗∗∗∗*p* value < 0.0001. (b) Western blot analysis of total lysate of WT, FAD cells treated or not with AFSC-EV, then revealed with the indicated primary antibodies. The graphs represent the mean ± SEM of densitometric analysis of 3 experiments, normalized to actin values. ^∗^*p* value < 0.05; ^∗∗^*p* value < 0.01; ^∗∗∗^*p* value < 0.001. (c) Western blot analysis of AFSC-extracellular vesicles derived from 4 donors, then revealed for CD9, CD81, and Rab5, as exosome markers, SOD1, and actin. The graph represents the mean ± SEM of densitometric analysis of 3 experiments, normalized to actin values. ∗*p* value < 0.05.

The analysis of ROS content shown in Figure 2(a) revealed that FAD neurons displayed a higher ROS level compared to WT cells, as expected. Conversely, GSH is lower in FAD neurons, even though not significantly. However, the exposure to AFSC-EV reduced the ROS content, while allowed the increase in GSH level.

Then, we investigated the expression of proteins related to ROS modulation, and WB analysis (Figure 2(b)) demonstrated that antioxidant enzymes, such as SOD1, thioredoxin reductases TrxR1 and TrxR2, and glutathione peroxidase 1 (Gpx1), were all more present in FAD+EV neurons than in FAD cells. The comparison between FAD and WT did not show differences, excepted for TrxR1. The increase in TrxR1 expression in FAD neurons could be related to an intracellular defense against the ROS rise that we previously observed.

In parallel, the FAD neurons displayed a higher level of Nox4, a ROS producing enzyme constitutively active. Indeed, it does not need cytosolic subunits for activation, though they are able to modulate Nox4 activity. This effect was reduced, at least in part, by the presence of EV. Meanwhile, no differences were observed for the expression of gp91phox, the typical subunit of Nox2, another NADPH oxidase expressed into the brain. Aside from phagocytic cells, including microglia, Nox2 is also found in endothelial cells and neurons [27].

Interestingly, the characterization of AFSC-EV revealed that all the samples, even though with different level of exosome markers (CD9, CD81 and Rab5), carried similar levels of SOD1 protein.

### 3.3. Effect of AFSC-EV on Apoptotic and Autophagic Pathways

**Figure 3:**
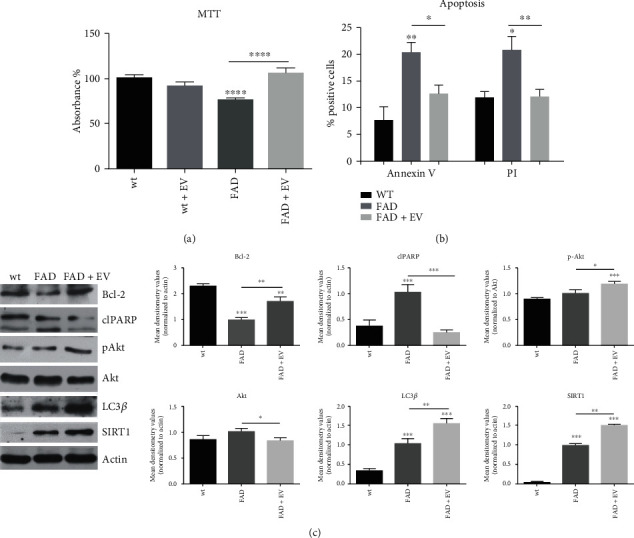
Effect of AFSC-EV supplementation on apoptotic and autophagic pathways of AD primary neurons *in vitro*. (a) Representative graph showing MTT viability of WT and FAD cells treated or not with AFSC-EV. ^∗∗∗∗^*p* value < 0.0001. (b) Representative graph showing the percentage of positive cells to Annexin V/Propidium Iodide assay, as described in the 2.12 section. ^∗^*p* value < 0.05; ^∗∗^*p* value < 0.01. (c) Western blot analysis of total lysate of WT, FAD cells treated or not with AFSC-EV, then revealed with anti-Bcl-2, anti-PARP, anti-pAkt, anti-LC3*β*, and anti-SIRT1 antibodies. The graphs represent the mean ± SEM of densitometric analysis of 3 experiments, normalized to actin values. ^∗^*p* value < 0.05; ^∗∗^*p* value < 0.01; ^∗∗∗^*p* value < 0.001.

Neuronal viability in FAD neurons was significantly decreased by 25% in DIV (days *in vitro*) 14 cultures, compared to WT neurons (Figure 3(a)). In parallel, the analysis of apoptosis by annexin V and propidium iodide (PI) demonstrated that the apoptotic pathway is involved, as expected (Figure 3(b)). However, the presence of EV significantly restored cell viability and reduced annexin V and PI staining (Figures 3(a) and 3(b)). In accordance with this result, our immunoblotting data further demonstrated an increased activation of Akt, a key signaling molecule for neuronal survival, indicated by the higher phosphorylation, with a concomitant activation of Bcl-2, an anti-apoptotic marker, in FAD+EV neurons (Figure 3(c)). Moreover, the cleavage of PARP, significantly higher in FAD neurons, compared to WT, was reduced by the EV-treatment. These results suggest that the decreased cell viability associated with the activation of apoptotic pathway in FAD neurons could be contained by AFSC-EV.

In order to test the role of the PI3K/Akt pathway, we used wortmannin, selective and irreversible inhibitor of phosphatidylinositol 3-kinase (PI3K). The results on ROS and apoptosis (Supplementary Figure I) show that the inhibition of the PI3K/Akt pathway partially affects the extracellular vesicle modulation, suggesting its involvement in the EV efficacy.

Autophagic pathways were also modulated through AFSC-EV content, as demonstrated by the increased levels of SIRT1, a NAD-dependent class III histone and nonhistone protein deacetylase, and LC3*β* protein. Both proteins are markers of autophagy, and their expression is altered during AD [28].

### 3.4. Reduction of AD Markers in the Presence of AFSC-EV

Finally we investigated the modulation of intracellular AD markers, by using the antibody 6E10, specific for human Amyloid precursor protein (APP), A*β* oligomers [29], and molecules generated from cleavage of APP by secretases [30], and anti-Tau phosphorylated in Serine 422 [31], a pathological epitope and one of the markers of neurofibrillary degeneration: both are neuropathological hallmarks of Alzheimer's disease.

**Figure 4:**
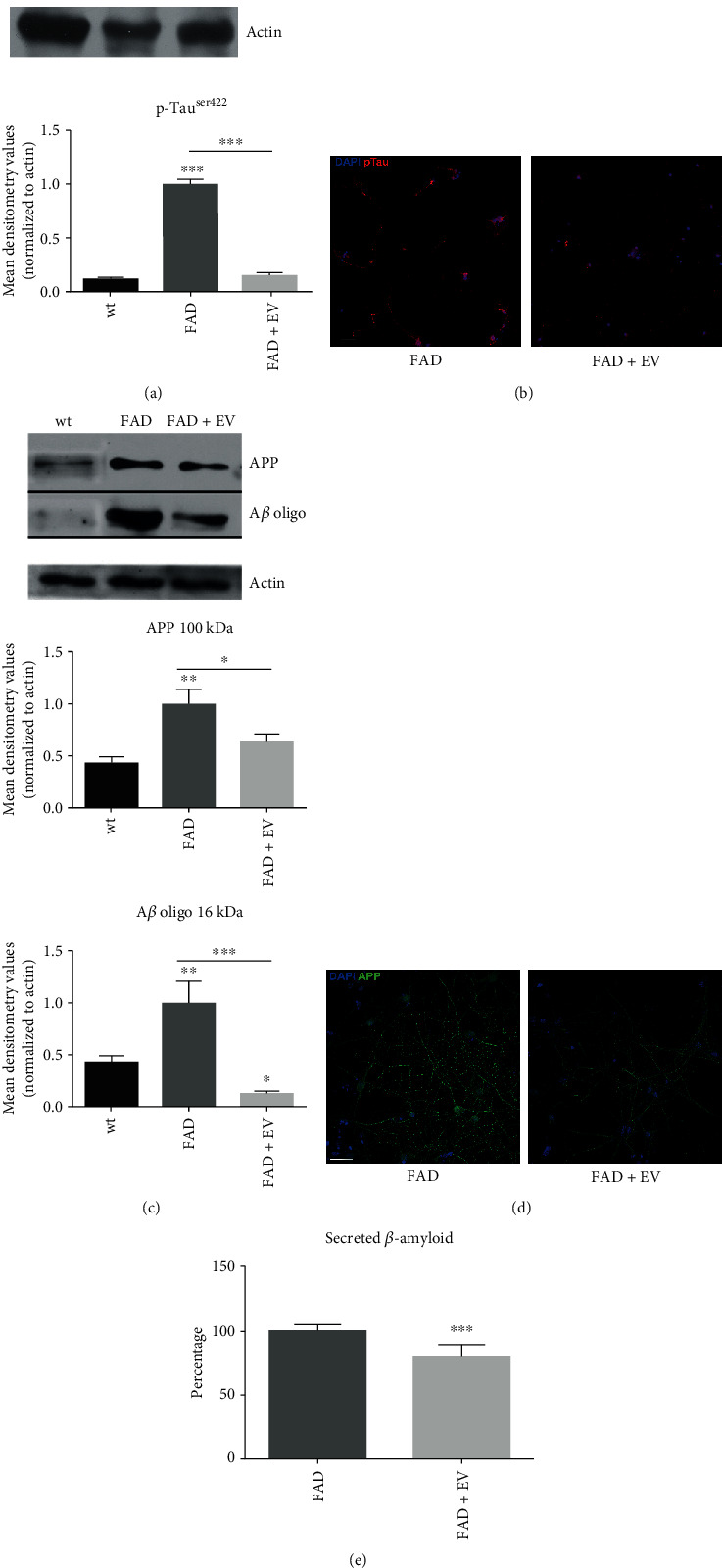
Effect of AFSC-EV supplementation on AD markers of AD primary neurons *in vitro.* (a) Western blot analysis of total lysate of WT and FAD cells treated or not with AFSC-EV, then revealed with anti-p-Tau^ser422^ antibody. The graph represents the mean ± SEM of densitometric analysis of 3 experiments, normalized to actin values. ^∗∗∗^*p* value < 0.001. (b) Representative images showing DAPI (blue) and p-Tau^ser422^ (red) signals of FAD cells incubated or not with EV. Scale bar = 10 *μ*m. (c) Western blot analysis of total lysate of WT, FAD cells treated or not with AFSC-EV, then revealed with 6E10 antibody. APP (100 kDa) and A*β* oligo (16 kDa) bands, with 6E10, are shown. The graphs represent the mean ± SEM of densitometric analysis of 3 experiments, normalized to actin values. ∗*p* value < 0.05; ^∗∗^*p* value <0.01; ∗∗∗p value<0.001. (d) Representative images with DAPI (blue) and APP (green) signals of FAD cells incubated or not with AFSC-EV are shown. Scale bar = 10 *μ*m. (e) Percentage of secreted *β*-amyloid of FAD cells treated or not with AFSC-EV for 7 days. These data are obtained by measuring the A*β*42 levels *via* ELISA as described in Section 2.9. ^∗∗∗^*p* value < 0.001.

Our immunoblotting results demonstrated that levels of APP, A*β* oligo, and p-Tau^ser422^ were reduced in EV-treated AD neurons (Figures 4(a) and 4(c)). These results were confirmed by immunofluorescence images shown in Figures 4(b) and 4(d), where the signals of 6E10 and p-Tau^ser422^ dropped down in EV-samples.

Furthermore, the quantitative detection of secreted A*β*42 demonstrated the positive effect of AFSC-extracellular vesicle on FAD neurons, since the production of A*β* in AD neurons was decreased after treatment.

## 4. Discussion

In the last decades, stem cells have been in the spotlight as a promising candidate for regenerative medicine. Amongst these, mesenchymal stromal cells from the amniotic fluid (AFSCs) have been shown to favor tissue repair and regeneration after transplantation in animal models of inflammatory-based diseases. The benefits of transplanted amniotic cells were observed despite cell engraftment in injured tissue, thus suggesting that these cells produce bioactive factors able to mediate the above-mentioned effects through paracrine signaling [32]. Stem cells secrete various molecules such as growth factors and extracellular vesicles [33], able to modulate oxidative stress. Since endogenous and microenvironmental oxidative stress may be a pivotal factor exacerbating pathways driving to neuronal loss in AD [34], here, we explored the effects of a suspension enriched in exosomes, derived from AFSCs in culture, on the viability of neurons obtained from AD mice, and demonstrated that the redox state is deeply modified by such exposure.

We are aware that the used isolation technique could give possible differences compared to ultracentrifugation and SEC protocols: indeed, it has been demonstrated that this kit gave the highest yield but the preparation showed broader size-distribution likely due to microvesicle co-precipitation and had the least dispersion stability [35]. Therefore, in order to compare the efficacy of these extracellular vesicles, obtained with this commercial kit, and the other part of secretome, namely, the supernatant derived from such isolation process (-EV), we performed viability and ROS analyses on the recognized in vitro AD model, SH-SY 5Y exposed to A*β*, treated with +EV or -EV. The results, shown in supplementary Figure II, confirmed our previous observations on other cell types (human lymphocytes) [15]: actually, the presence of EV showed an increase in the viability of A*β*-treated cells and a decrease in ROS content, but - EV did not exert any modulation, supporting the validity of the extracellular vesicle effect on neurons obtained from AD mice.

Such modulation, described above, occurred in parallel with a decrease in the accumulation of extracellular amyloid-*β*. Accordingly, the key biochemical change in AD is the modification by the ROS of A*β* into toxic products, which progressively aggregate into senile plaques promoting apoptosis [36]. Indeed, considerable evidence now indicates that soluble A*β* oligomers, monomers, and protofibrils, rather than amyloid deposits, are the main toxic agents in AD [37]. Consistently, we observed a decrease in the APP and A*β* oligomers intracellular content as well.

In line with this consideration, we noticed that the rescue of neurons viability exposed to AFSC-EV is accompanied by a decline in apoptotic markers. Moreover, morphological aspects, deriving from neurotoxicity, such as neurite and nuclear envelope modifications, were prevented. Typical nuclear abnormalities consisting of altered nuclear envelope/lamina structure were observed in several age-related diseases [38] and in AD as well [39].

Other AD features were also taken into consideration, such as impairment of autophagy and Tau phosphorylation, since both of them are, to a certain extent, linked to redox unbalance. Indeed, oxidative stress also supports a process of progressive failure of autophagy in neurons and promotes Tau phosphorylation, then causing impaired memory deficit [1]. Moreover, growing evidence suggests that APP processing and A*β* release are upstream of Tau pathology. Tau hyperphosphorylation is implicated in autophagy dysfunction, and increased levels of lysosomal protease occur in AD patients [40]. Indeed, we noticed that FAD samples showed a higher level of LC3B, the protein involved in the formation of autophagosomal vacuoles, an event probably due to an early cellular response to the need to eliminate protein aggregates, which remains insufficient to counteract the progress of the pathology. More interestingly, we observed that the presence of extracellular vesicle treatment induced a further increase in the autophagic marker LC3B. EV administration positively regulated SIRT1 expression as well. This effect supports the proposed role of extracellular vesicles in regulating ROS levels and autophagy, since sirtuins are antioxidant and antiapoptotic and crucial mediators for lysosomal autophagy regulation, which plays a pivotal role in the AD [41].

Moreover, ROS has been shown to regulate autophagy through several signaling pathways such as PI3K/Akt, AMPK. These pathways appear to be crucial in AD since related to the Tau protein hyperphosphorylation. In addition, the PI3K/Akt pathway has been shown to play a critical role in the neuroprotection and inhibiting apoptosis via the enhancing expression of the SODs [42]. Similarly, here, we demonstrated that the presence of extracellular vesicles favored the activation of Akt, being this protein more phosphorylated after EV-exposure. Moreover, in case of inhibition of the PI3K/Akt pathway, the extracellular vesicle modulation ROS and apoptosis was affected, suggesting its partial involvement in the EV efficacy. All these data are in line with the observations carried out on a model of AD, generated by treating *in vitro* SH-SY 5Y cells (a human neuroblastoma cell line) with A*β*, where the authors showed that BM-MSCs stimulated autophagy in diseased neurons exposed to toxic protein aggregates and the enhancement of aggregate degradation, thus increasing neuronal survival [43].

Interestingly, we then confirmed that the treatment with AFSC-extracellular vesicles prevented the phosphorylation of Tau protein at serine 422, the typical site of phosphorylation occurring in AD [31], suggesting that the redox regulation by extracellular vesicles may affect the whole AD pathogenesis. This is consistent with the hypothesis that increased ROS production may have an integral role in the development of sporadic AD prior to the appearance of amyloid and Tau pathology [44].

The mechanisms proposed by de Godoy et al. [14] to explain, at least in part, neuroprotection by MSC-EVs pertains to the content in antioxidant enzymes and in anti-inflammatory and/or trophic molecules. The authors demonstrated that EVs secreted by MSCs contain and carry endogenous catalase that endows EVs of ROS scavenging activity. Similarly, we clearly observed that SOD1 is contained into AFSC-extracellular vesicles. SOD1 presence might be one of the mechanisms of protection exerted by extracellular vesicles in neurodegeneration, avoiding an excess of reactive oxygen species. A decrease in reduced glutathione (GSH) causes excess in ROS production leading to oxidative stress, thus favoring AD pathogenesis [45]. Here, we demonstrated that exposure to AFSC-EV induced a significant rise in glutathione peroxidase (Gpx) enzyme; thus, it is not surprising to see that EV-treatment increased GSH levels in FAD neurons. These samples showed a slightly minor content of GSH, compared to WT, in accordance with the data obtained by Resende et al. in the 3xTg-AD mice, an AD mouse model, where decreased levels of GSH were observed [46]. A similar trend, even if not statistically significant, also occurred in WT cells, when treated with EV. This slight difference between WT and FAD neurons in GSH levels after 7 days in culture is likely to be due to a cellular defense that can be noticed in the expression increase of the antioxidant enzyme thioredoxin reductase 1 (TrxR1). Moreover, both TrxR1 and TrxR2 presence surged after EV-exposure, contributing with SOD1 and Gpx to the ROS decrease observed in these samples. This complex redox balance is also influenced by the presence of prooxidant enzymes, namely NADPH oxidases. In this experimental condition, Nox4, the isoform constitutively active, showed a higher content in FAD samples, being reduced, at least partially, after EV-treatment. Regarding gp91phox, the specific subunit of Nox2, no differences could be observed, perhaps because the activation of this isoform derives mostly from a regulating subunit interaction [27].

The analysis of our data demonstrated that extracellular vesicles derived from stem cells have a direct effect on neurons affected by AD, modulating ROS content. Meanwhile, most of the literature shows an indirect therapeutic potential, underlining a regulation of neuroinflammation reducing microglial activation [47] [48] [49].

We have previously demonstrated that AFSC-extracellular vesicles contain anti-inflammatory molecules, namely, HFG, TGF*β,* and pentraxin 3 [15] that showed a therapeutic effect on osteoarthritis [16]. Even in this context, pentraxin 3, that is, a protein regulator of angiogenesis and neurogenesis with direct involvement in neuroinflammation in acute phases [50], could have a therapeutic role. The positive effect of MSC secretome on both fronts, namely, neurons and microglial cells, could be the winning key that justifies the encouraging results obtained on AD features by using MSCs *in vivo*. For example, the transplantation of human umbilical cord blood and adipose-derived MSC, into the hippocampus of mouse AD models, reduced A*β* plaques, increased endogenous adult neurogenesis and synaptic activity, and enhanced cognitive function [51] [52].

## 5. Conclusions

In conclusion, in this study, we focused our attention on extracellular vesicles in preventing the disease phenotypes of the only neurons in culture. We employed cells deriving from 5xFAD mice [53], a more complete animal model of AD, if compared to Tg2576 from which primary neurons were obtained in the study by Lee et al. [54]. Indeed, Tg2576 is a mouse model carrying a single human amyloid precursor protein mutation (APPswe) [55]. However, in our model, the exposure of MSC extracellular vesicles ameliorated the progression of A*β*-induced neuronal death and AD as well, supporting the idea proposed by Lee et al. that extracellular vesicles, derived in that case from adipose tissue, could exert disease-modulating effects [54]. Furthermore, here, we clearly demonstrated a ROS regulation involvement in extracellular vesicle therapeutic effect in neurodegenerative diseases, such as AD.

## Data Availability

Previously reported electron microscopy data were used to support this study and are available at doi:10.1002/biof.1407. These prior studies (and datasets) are cited at relevant places within the text as references [15].

## Abbreviations

AFSCs: Amniotic fluid stem cells
BSA: Bovine serum albumin
DCFH-DA: Dichlorodihydrofluorescein diacetate
DAPI: 4′,6-diamidino-2-phenylindole
EDTA: Ethylenediaminetetraacetic acid
EV: Extracellular vesicles
PBS: Phosphate buffered saline
HEPES: 4-(2-hydroxyethyl)-1-piperazineethanesulfonic acid
MCB: Monochlorobimane
SDS: Sodium dodecyl sulfate
WB: Western blot.

## Supplementary Materials

Supplementary Materials Supplementary Figure I: effect of Inhibition of P13K/Akt on apoptosis and ROS of FAD neurons. Supplementary Figure II: effect of hAFSC secretome on AB-induced neuron toxicity and ROS level.
