# Supplementary material for: Oxidative Stress in Alzheimer's Disease: In Vitro Therapeutic Effect of Amniotic Fluid Stem Cells Extracellular Vesicles
Source: Oxid Med Cell Longev. 2020 Oct 24;2020:2785343. doi: 10.1155/2020/2785343 (PMC7641262; doi:10.1155/2020/2785343)
Supplement: Supplementary Materials — Supplementary Figure I: effect of Inhibition of P13K/Akt on apoptosis and ROS of FAD neurons. Supplementary Figure II: effect of hAFSC secretome on AB-induced neuron toxicity and ROS level. [file 2785343.f1.pdf]

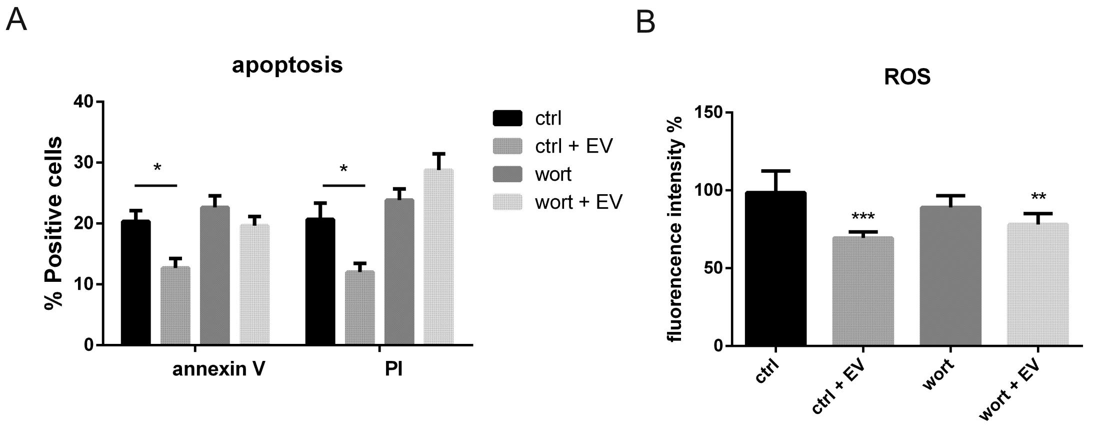

Supplementary figure I - Effect of inhibition of PI3K/Akt on apoptosis and ROS of FAD neurons.

FAD primary neurons (4 DIC) were treated with 150 nM wortmannin for 2 h before the exposure to EV and ROS and apoptosis assays were performed after 4 days.

A - Representative graph showing the percentage of positive cells to AnnexinV/Propidium Iodide assay, as described in the 2.12 section. *p value<0.05.

B - ROS content was measured with fluorescent probe, as described in 2.8 section, after 4 days of EV exposure. **p value<0.01; ***p value <0.001.

A

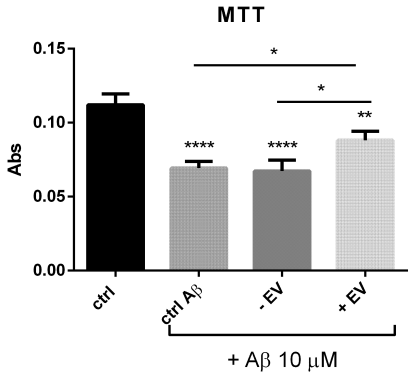

B

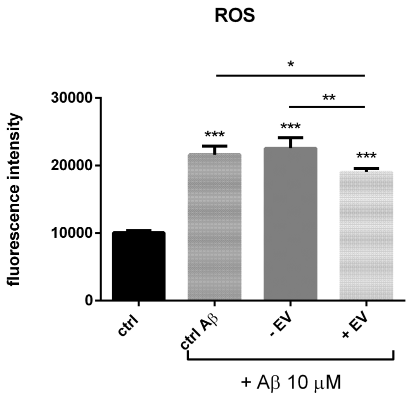

Supplementary figure II - Effect of the hAFSC secretome on Aβ- induced neuron toxicity and ROS level.

The neuroblastoma cell line SH- SY 5Y were exposed to 10 μM Aβ 1-42 for 24 h. The same protein concentration of + EV and – EV, obtained as described in 2.3 section, was added to neuronal cells 3 days before Aβ treatment. MTT (A) and ROS (B) assays were then performed. The graphs represent the mean of 3 experiments ± SEM of absorbance and fluorescence intensity, respectively. *p value<0.05; **p value<0.01; ***p value<0.001; ****p value<0.0001.
